# Metal–Ligand Coordination Nanomaterials Tailored to the Cancer-Immunity Cycle

**DOI:** 10.1021/acs.accounts.6c00430

**Published:** 2026-10-21

**Authors:** Peng Bao, Bei Li, Yunlu Dai

**Affiliations:** † Faculty of Medicine, 59193 University of Macau, Taipa, Macau SAR 999078, China; ‡ MoE Frontiers Science Center for Precision Oncology, 59193 University of Macau, Taipa, Macau SAR 999078, China

## Abstract

The successful implementation of antitumor immunity hinges largely on a chain of stepwise events known as the cancer-immunity cycle (CIC). In this cycle, tumor cell death caused by T lymphocytes triggers inflammatory rounds of antigen-presenting cell (APC)-mediated antigen presentation and T-cell stimulation, sustaining active tumoricidal immunity, whereas tumor immunosuppressive networks impose multifaceted detrimental effects on essential elements involved in the CIC, which are considered therapeutic targets for improved tumor immunotherapy. Tremendous evidence has thrown light on the pivotal roles of metal ions in oncology, especially in antitumor immunity. Metal ions participate in modulating the step-by-step events occurring in the CIC, principally via their structural, catalytic, or modulatory interactions with immunosensors, ion transporters, and downstream effectors, as exemplified by Mn^2+^ that sensitizes cyclic GMP–AMP synthase and the stimulator of interferon genes pathway to trigger antitumor T-cell immunity. Owing to their lack of targeting ability, metal ions used alone cannot serve as immunotherapeutics. Inspired by the excellent targeting properties of nanomedicines and the unique ability of metal ions to self-assemble with diverse functional ligands, recent decades have witnessed rapid advances in metal–ligand coordination nanomaterials for enhanced tumor immunotherapy. Unlike other nanomedicines, metal coordination complexes can serve as multifunctional immunotherapeutic platforms, due to their chemical tunability and design versatility. Their unique composition and structure allow them to exhibit substantial outstanding properties, blazing the promising trail of tumor immunotherapy.

In this Account, we summarize our recent breakthroughs in the development of metal–ligand coordination nanomedicines, especially in the therapeutic mechanisms tailored for CIC, with the ultimate purpose of ameliorative cancer immunotherapy. These promising nanomedicines are designed on the basis of stepwise events within the CIC, in which the targets can be subdivided into (1) tumor cells, (2) APCs, and (3) T cells. Once targeted to tumor cells, these nanomaterials generally induce immunogenic tumor cell death, leading to the release of numerous danger-associated molecular patterns that exert immune-stimulating effects on the APCs. Metal coordination complexes also directly activate APCs by the intrinsic immunostimulatory properties of metal ions or by delivering other adjuvants. Both pathways promote APC activation and antigen presentation for the subsequent priming of antitumor T-cell responses. Apart from indirect APC-to-T immunostimulatory pathways, these nanomedicines directly act on T cells and reinforce their antitumor functions, including activation, trafficking, infiltration, and tumor-killing capabilities. In addition, both future challenges and the anticipated development blueprint of these medicines as clinical therapeutics are emphasized.

## Graphical abstract

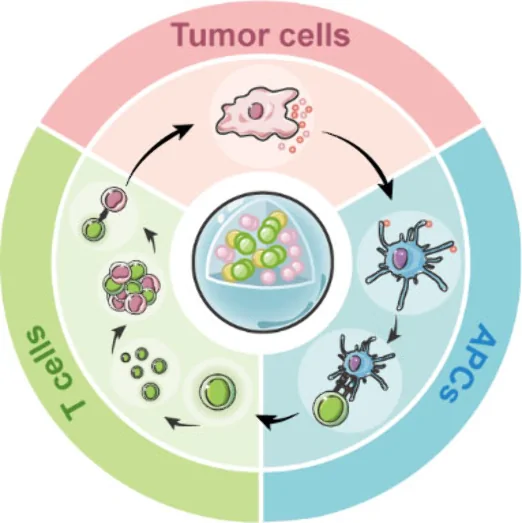

## KEY REFERENCES

-

Li, B. ; Wang, G. ; Miao, K. ; Zhang, A. ; Sun, L. ; Yu, X. ; Lei, J. H. ; Xie, L. ; Yan, J. ; Li, W. ; Deng, C. ; Dai, Y. Fueling sentinel node via reshaping cytotoxic T lymphocytes with a flex-patch for post-operative immuno-adjuvant therapy. Nat. Commun. 2023, 14, 2518, DOI: 10.1038/s41467-023-38245-7 PMC1015442137130873.^1^ A postoperative flex-patch that integrated magnesium iron-based layered double hydroxides (LDHs) and PD-1 antibody was devised to revitalize cytotoxic T cells and memory T cells within sentinel lymph nodes, allowing for the sustained surveillance against postsurgical cancer recurrence.
-

Huang, Z. ; Huang, S. ; Song, S. ; Ding, Y. ; Zhou, H. ; Zhang, S. ; Weng, L. ; Zhang, Y. ; Hu, Y. ; Yuan, A. ; Dai, Y. ; Luo, Z. ; Wang, L. Two-dimensional coordination risedronate-manganese nanobelts as adjuvant for cancer radiotherapy and immunotherapy. Nat. Commun. 2024, 15, 8692, DOI: 10.1038/s41467-024-53084-w PMC1145876539375342.^2^ A risedronate-manganese nanocoordinator was constructed to elicit potent ICD and further reinforce in situ cancer vaccination, as a synergistic enhancer for tumor radiotherapy and immunotherapy.
-

Wang, G. ; Xie, L. ; Li, B. ; Sang, W. ; Yan, J. ; Li, J. ; Tian, H. ; Li, W. ; Zhang, Z. ; Tian, Y. ; Dai, Y. A nanounit strategy reverses immune suppression of exosomal PD-L1 and is associated with enhanced ferroptosis. Nat. Commun. 2021, 12, 5733, DOI: 10.1038/s41467-021-25990-w PMC848426134593794.^3^ An immunotherapeutic nanounit codelivering an exosome inhibitor and Fe^3+^ was fabricated to suppress the PD-L1-driven immune resistance of tumor-derived exosomes and amplify tumor cellular ferroptosis, unleashing the tumoricidal potential of cytotoxic T cells.
-

Xie, L. ; Li, J. ; Wang, G. ; Sang, W. ; Xu, M. ; Li, W. ; Yan, J. ; Li, B. ; Zhang, Z. ; Zhao, Q. ; Yuan, Z. ; Fan, Q. ; Dai, Y. Phototheranostic Metal-Phenolic Networks with Antiexosomal PD-L1 Enhanced Ferroptosis for Synergistic Immunotherapy. J. Am. Chem. Soc. 2022, 144, 787–797, DOI: 10.1021/jacs.1c09753 34985903.^4^ A phototheranostic metal-phenolic network that integrated an exosome inhibitor and Fe^3+^ was prepared to counteract the immunosuppression actuated by tumor-shed exosomes, boost photothermal efficacy, and expedite tumor cell ferroptosis, culminating in strengthened antitumor immunity.

## 1 Introduction

Cancer immunotherapy, which harnesses the autologous immune system to mount a devastating assault against malignant cells, has emerged as a cornerstone of cancer treatment.^5,6^ The successful development of antitumor immunity is largely contingent upon the potent cytotoxic T-cell response, which in turn depends on a cascade of stepwise events collectively known as the cancer immunity cycle (CIC).^7^ This cycle is initiated with the immunogenic death of tumor cells, during which released tumor antigens are captured, processed, and presented by antigen-presenting cells (APCs) to naive T cells, culminating in T-cell priming and activation.^8^ The resulting effector T lymphocytes subsequently traffic through the circulation, extravasate across the vascular endothelium, and infiltrate the tumor bed, where they execute tumoricidal functions that trigger a new round of CIC through the liberation of additional tumor antigens and danger signals. Given that each node of this cycle is systematically suppressed by the immunosuppressive tumor microenvironment (TME), such as spanning hypoimmunogenic tumor phenotypes, defective APC maturation, and impaired T-cell effector functions, a central imperative in cancer immunotherapy is the rational design of therapeutics capable of selectively targeting and reinvigorating these compromised elements of the CIC.

Mounting evidence has established that metal ions play pivotal roles in modulating discrete events within the CIC through their coordination interactions with biological ligands.^9^ Mn^2+^ enhances the sensitivity of cyclic GMP–AMP synthase (cGAS)-stimulator of interferon genes (STING) pathway by promoting dsDNA-induced liquid-phase condensation of cGAS and facilitating STING oligomerization, triggering a multifaceted pro-inflammatory program that propels APC activation and antigen presentation.^10^ Mg^2+^ amplifies T-cell receptor (TCR)-proximal signaling via lymphocyte function-associated antigen 1 (LFA-1)-mediated sensing and downstream focal adhesion kinase (FAK) and extracellular signal-regulated protein kinases 1/2 (ERK1/2) cascades to reinforce T-cell effector functions,^11^ while Zn^2+^ stabilizes the Lck-CD4/CD8 coreceptor complex through a zinc-clasp structural scaffold.^12^ K^+^ homeostasis further preserves T-cell stemness by preventing exhaustion-associated epigenetic reprogramming.^13^ These immunomodulatory functions provide a compelling blueprint for metal-based cancer immunotherapy. However, free metal ions lack tumor-targeting specificity and cause severe off-target toxicity, limiting their direct clinical translation. The convergence of nanomedicine and coordination chemistry has addressed this challenge through metal–ligand coordination nanomaterials, a class of inorganic–organic hybrid platforms formed by the self-assembly of metal ions with rationally designed organic ligands (e.g., polyphenols, catechols, phosphonates, and functional polymers).^14^ Unlike conventional nanocarriers that function primarily as passive cargo vehicles, these coordination complexes are inherently bioactive. Their metal centers simultaneously serve as structural nodes that direct supramolecular architecture and as functional entities that execute metal-specific therapeutic programs.^15^ Moreover, their modular coordination chemistry enables the seamless integration of diverse functional components, including photosensitizers, immune adjuvants, metabolic inhibitors, and epigenetic modulators, into a single nanosystem with tunable stimulus-responsive properties.^16,17^ This design versatility empowers metal–ligand coordination nanomaterials to serve as potent inducers of immunogenic tumor cell death, as immunoadjuvants that directly activate APCs, or as sensitizers that amplify antitumor T-cell immunity.

Despite these advances, a systematic framework that maps the therapeutic mechanisms of metal–ligand coordination nanomaterials onto the sequential events of the CIC remains lacking. Most extant reviews have focused primarily on the biomedical applications of these materials in drug delivery, with only cursory treatment of their immunotherapeutic mechanisms.^14–17^ In this Account, we present a unified perspective on our recent efforts in elucidating and engineering metal–ligand coordination nanomaterials as CIC-tailored immunotherapeutics (Scheme 1). These nanotherapeutics are designed to reinvigorate antitumor immunity via three principal pathways corresponding to the key cellular targets of the CIC: (1) targeting tumor cells to enhance their immunogenicity via immunogenic cell death (ICD) and immune checkpoint blockade (ICB); (2) potentiating APC activation and antigen presentation through STING pathway engagement and phagosomal modulation; and (3) reinforcing T-cell antitumor functions, including activation, trafficking, infiltration, and sustained cytotoxicity. We also critically assess the outstanding challenges impeding clinical translation, such as metal-ion-specific toxicity, pharmacokinetic complexity, and manufacturing scalability, and outline future directions for next-generation coordination-based cancer immunotherapeutics. Through this Account, we aim to provide a conceptual framework for the rational design of metal–ligand coordination nanomaterials that precisely interrogate and amplify each step of the CIC, accelerating the development of tailored metalloimmunotherapies with improved clinical outcomes.

**Scheme 1:**
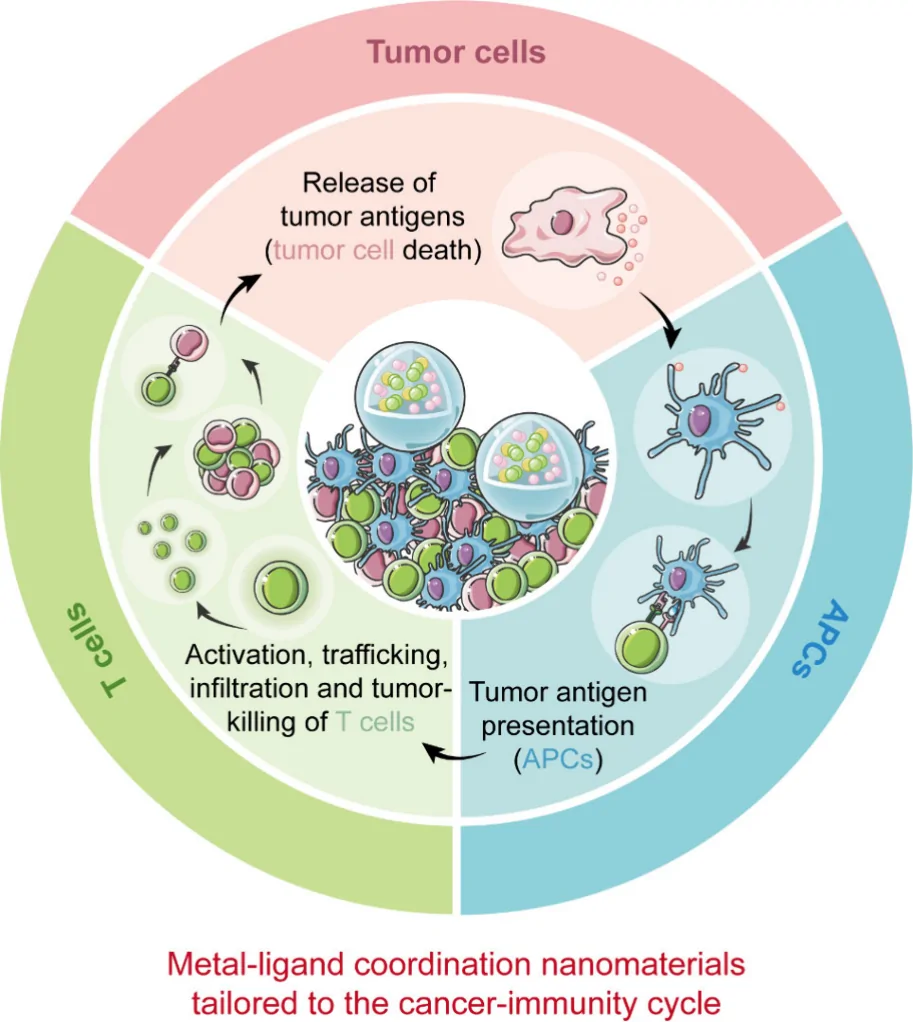
Schematic Illustration of Design Principles for Metal–Ligand Coordination Nanotherapeutics Tailored to the Step-by-Step Events Involved in the CIC^a^

a These nanotherapeutics rejuvenate anti-tumor immunity through three mechanistic axes that align with the core cellular nodes of the CIC: (1) boosting tumor immunogenicity via ICD and ICB; (2) enhancing APC activation and antigen presentation through STING pathway engagement and phagosomal remodeling; (3) strengthening T-cell anti-tumor activity, including activation, intratumoral trafficking, infiltration, and persistent cytotoxicity.

## 2 Targeting Tumor Cells

Tumor cells resort to a variety of cunning tricks for evading immune-mediated obliteration by acquiring manifold characteristics that undermine antitumor immunity, ranging from the activation of APCs to the implementation of T-cell effector functionality.^18,19^ During tumor evolution and development, tumor cells undergo persistent alterations and reinforcement under immune-resistant pressure, leading to the clonal selection of hypoimmunogenic subsets.^20^ These undesirable subclones are insusceptible to the CIC-driven antitumor immune response, even after their death. Encouraged by the preponderance of metal coordination complexes in tumor immunotherapy, diverse treatment tactics utilizing these nanomedicines have been proposed to target tumor cells and enhance their immunogenicity.

### 2.1 Inducing ICD

Unlike physiological apoptosis accompanied by immune silencing, ICD is a specific pattern of cell death that converts dying hypoimmunogenic tumor cells into in situ-generated cancer vaccines, actively initiating and amplifying the CIC-driven antitumor immunity.^21,22^ Once ICD occurs, dying tumor cells can release large amounts of tumor antigens and immunostimulatory damage-associated molecular patterns (DAMPs).^23^ These signal molecules serve as adjuvants to evoke a CIC-enhancing process that activates APCs and assists antigen presentation for strengthening antitumor T-cell response.

Over recent decades, extensive research has been engaged in the development of nanotherapeutics designed to trigger ICD.^24–26^ While promising, conventional ICD-triggered nanomedicines frequently fail to conquer the complex immunosuppressive TME.^23^ Differentiated from traditional drug-delivery nanoplatforms that hinge on their payload, the modular design of metal–ligand coordination nanomaterials integrates the cellular homeostasis-disrupting properties of metal ions with the multifaceted functionality of ligands. This design allows for efficient induction of ICD through diversified modalities such as ferroptosis, cuproptosis, pyroptosis, or their combinations.^17^

Building on this concept, our team devised a versatile copper-piceatannol/hyaluronic acid nanopill (Cu-Pic/HA NP) that elicited both cuproptosis and pyroptosis through comprehensive polyamines depletion, eliciting a potent and sustained antitumor immunity (Figure 1a).^27^ The nanopill was synthesized through one-pot coordination-driven self-assembly of Cu^2+^ and piceatannol, followed by hyaluronic acid surface modification. Since the metal center functions simultaneously as the framework node and the bioactive effector, the feed ratio of metal to ligand offers a direct lever for programming the relative weighting of cuproptosis versus pyroptosis. To achieve an optimal formulation, our group adopted a systematic optimization strategy. Specifically, we synthesized Cu-Pic nanoparticles across a range of Cu^2+^ to piceatannol feeding ratios and evaluated key parameters including particle size, colloidal stability, Cu^2+^ loading capacity, and release profiles. The optimal ratio was selected on the basis of comprehensive characterization to ensure that the resulting nanoparticles exhibited both high copper loading and sufficient piceatannol retention for comprehensive polyamine depletion. Significantly, the all-around elimination of polyamines was achieved by restraining their synthesis, obliterating existing stores and blocking uptake, leading to cellular redox imbalance and mitochondrial dysfunction. This disorder further precipitated the accumulation of Cu^2+^ within mitochondria, which induced the aggregation of toxic thioacylated dihydrothiamide S-acetyltransferase (DLAT) proteins for the subsequent induction of cuproptosis. In addition, complete removal of intracellular polyamines evoked a surge in mitochondrial reactive oxygen species (ROS) levels. This, in turn, reinforced the expression of zinc finger DHHC-type palmitoyltransferase 5 (zDHHC5) and zDHHC9 to aggrandize gasdermin D (GSDMD) palmitoylation, with the ultimate goal of enhanced pyroptosis. Both cuproptosis and pyroptosis were immunogenic modalities that culminated in ICD, collectively eliciting the release of canonical DAMPs, including surface-exposed calreticulin (CRT), secreted adenosine triphosphate (ATP), and high-mobility group box 1 (HMGB1), together with abundant tumor antigens (Figure 1b). These danger signals reshaped the immune-resistant TME and boosted the antineoplastic immune responses. This desired antitumor immunity dealt a devastating blow to tumor cells, yielding the prominent therapeutic benefits of inhibiting tumor proliferation (Figure 1c,d). Beyond cuproptosis and pyroptosis, we also engineered an amifostine-iron nanoparacrystalline (AFe-NPC) system to selectively induce ferroptosis in tumors with high alkaline phosphatase (ALP) expression.^28^ AFe-NPC-triggered ferroptosis similarly induced ICD, with the release of DAMPs and tumor antigens that actuated the CIC. This also exerted a synergistic effect with immune checkpoint inhibitors, thereby exterminating both the primary and metastatic tumor lesions.

**Figure 1:**
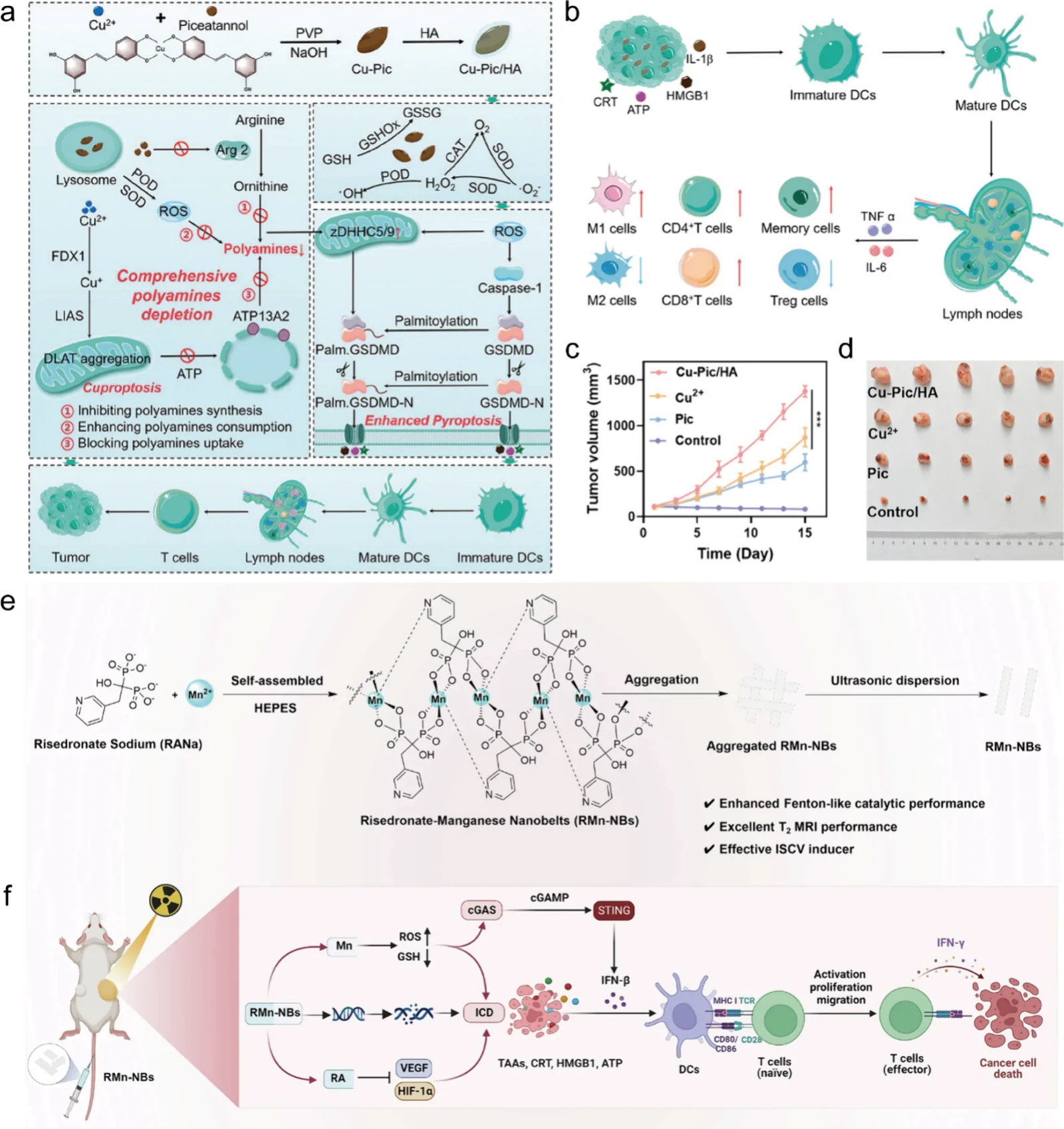
Immunotherapeutic tactics for inducing ICD of tumor cells. (a) Schematic illustration of Cu-Pic/HA NP-evoked immunogenic cuproptosis and pyroptosis. (b) Therapeutic mechanisms of Cu-Pic/HA NP-mediated antitumor immune response. (c) Tumor growth curves of mice. (d) Tumor images of mice. Reproduced with permission from ref 27. Copyright 2024 Wiley-VCH. (e) Design and fabrication of RMn-NB. (f) Schematically illustrating the strategy of RMn-NB-sensitized RT for triggering generation of potent in situ cancer vaccination. Reproduced with permission from ref 2. Copyright 2024 Springer Nature.

In addition to ICD-inducing nanotherapeutics, high atomic number (high-Z) metal-containing radiosensitizers enhance localized X-ray energy deposition, which thus increases DNA damage and improves radiotherapy (RT) efficacy at an equivalent radiation dose.^29^ Guided by this rationale, we developed a battery of high-Z metal coordination nanosensitizers for revitalizing RT-mediated ICD.^30–32^ As an illustration, a metal-phenolic RT sensitization nanoplatform (Hf-PSP-DTC@PLX) was constructed via integration of Hf-chelated phenolic semiconductive polymer (Hf-PSP), hydrogen sulfide (H_2_S) donor (polyethylene glycol-*co*-polydithiocarbamates, PEG-DTC), and amphiphilic polymer (poloxamer F127, PLX).^30^ Drawing parallels with the concept articulated previously, an oxygen metabolism-reprogramming tactic was devised to circumvent RT resistance that was actuated by intrinsic tumor hypoxia. The resulting H_2_S undermined mitochondrial respiration to curtail the intracellular oxygen consumption. The well-preserved oxygen fostered Hf-provoked radiosensitization, which consequently improved ICD-driven antineoplastic immunity. Likewise, our team designed a RT-empowered nanoplatform (RMn-NB) that was constructed through coordination-directed assembly of risedronate and Mn^2+^ for enhanced in situ cancer vaccination (Figure 1e).^2^ This nanoplatform sensitized RT through inhibiting the hypoxia-inducible factor 1α (HIF-1α)/vascular endothelial growth factor (VEGF) axis (Figure 1f). Leveraging Fenton-like catalytic activity conferred by Mn^2+^, RMn-NB potently elicited ICD, accompanied by the release of abundant DAMPs (e.g., CRT, ATP, HMGB1) and tumor antigens, thereby orchestrating CIC-augmented antitumor immunity. Moreover, we developed a tungsten-based nanocoordinator (PWE) that combined W^6+^ radiosensitization with epigenetically modulated pyroptosis.^31^ Epigallocatechin-3-gallate (EGCG), a phenolic DNA methyltransferase inhibitor, restored GSDME expression, and concurrently caspase-3 activation by W^6+^ radiosensitization cleaved GSDME into its N-terminal fragment, launching radiotherapeutic pyroptosis-triggered CIC.

### 2.2 Blockading Immune Checkpoints

Recent decades have witnessed unprecedented advances in ICB therapy, which have initiated a new age in the realm of tumor precision immunotherapy.^33^ Immune checkpoint inhibitors are devised for intercepting the endogenous suppressive signals that constrain T-cell activation, as exemplified by the blockade of the programmed cell death 1 (PD-1)/programmed death ligand-1 (PD-L1) axis, potently unleashing and sustaining antitumor T-cell immunity.^34^ Despite promising results, ICB therapy remains encumbered by significant clinical obstacles as only a limited fraction of patients experience durable remission and long-term disease control.

To address these limitations, our team has extensively investigated multifunctional metal coordination nanoplatforms as synergistic agents for enhanced therapeutic effects of ICB therapy.^35–37^ For instance, we put forward a three-pronged antineoplastic regimen utilizing a PD-L1 antibody (aPD-L1)-modified platinum@polymer-catechol (APTP) nanobraker (Figure 2a).^35^ This nanoplatform was synthesized by oxidizing radiosensitizing Pt^2+^ to Pt^0^ via an amphiphilic PEG-catechol derivative, incorporating mammalian target of rapamycin (mTOR) suppressor (TAK228), and subsequently adsorbing aPD-L1 onto the surface. The conjugated aPD-L1 and released TAK228 disrupted PD-1/PD-L1 axis through direct blockade of PD-L1 ligands and indirect inhibition of protumorigenic mTOR signaling pathway (Figure 2b). Significantly, high-Z Pt combined with TAK228 yielded the distinctly ameliorative outcome of RT, leading to accelerated cell death. This combination strategy exerted a potent and systematic tumoricidal immunity, as evidenced by the dramatic abrogation of primary lesion progression and the concurrent retardation of distant tumor expansion (Figure 2c–e). Besides, we developed a repurposing strategy for existing drugs tailored to distinct downregulation of PD-L1 expression.^36^ A self-degradable nanogel (PMI) was designed to integrate metformin and imiquimod into a phenolic polymer. Leveraging metformin-stimulated PD-L1 degradation, PMI nanogel reversed the immunosuppressive tumor immune landscape into tumoricidal states.

**Figure 2:**
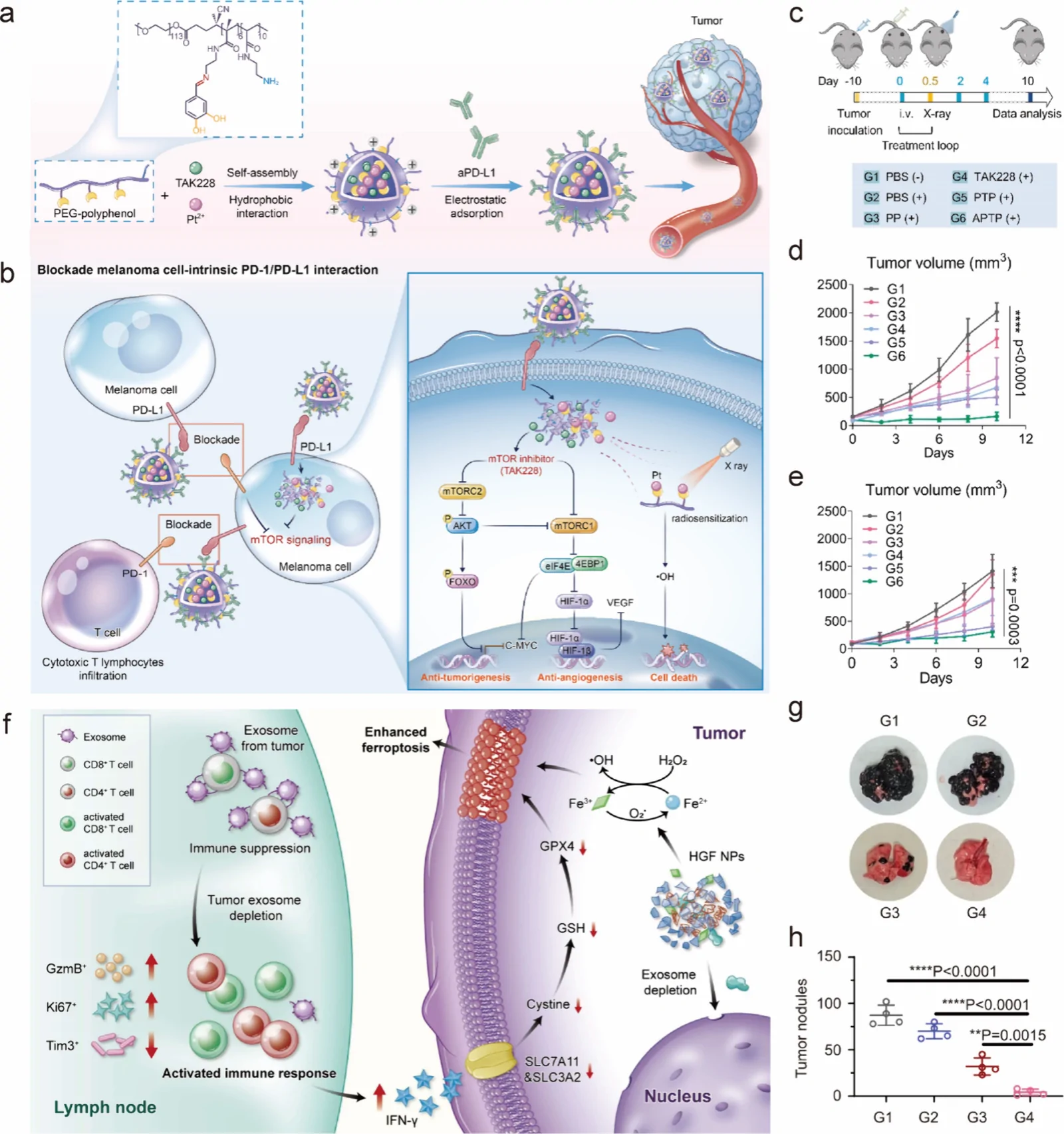
Design strategies for tumor immune checkpoint blockade. (a) Formulation and preparation of APTP nanobraker. (b) Immunotherapeutic mechanism illustration of APTP NP-mediated tumoricidal effect through blockading PD-1/PD-L1 pathway. (c) Schematically illustrating tumor therapeutic procedure in a murine B16F10 melanoma model. (d) Primary and (e) distant tumor growth curves of mice. Reproduced with permission from ref 35. Copyright 2023 Elsevier. (f) Schematic exhibiting the mechanism of HGF NP-evoked antitumor immunity via restraining the secretion of tumor-originated exosomes. (g) Representative images and (h) number statistics of metastatic nodules in the lungs of mice. G1: PBS; G2: anti-PD-L1; G3: HGF NP; G4: HGF NP combined with anti-PD-L1. Reproduced with permission from ref 3. Copyright 2021 Springer Nature.

Beyond surface PD-L1 expression, tumor cells secrete PD-L1-enriched exosomes to derange the antitumor functions of T cells, resulting in resistance to ICB therapy.^38^ In this context, we formulated pioneering strategies centered on exosomal PD-L1 blockade, achieved by developing nanoplatforms that abrogated the shedding of tumor-derived exosomes.^3,4^ An immunotherapeutic nanounit (HGF NP) was fabricated through the integration of exosome inhibitor (GW4869) into a prefunctionalized nanocarrier, which was prepared via coordinating the ferroptosis inducer (Fe^3+^) onto amphiphilic HA polymer (Figure 2f).^3^ Profiting from GW4869-driven inhibitory effect on biogenesis and release of tumor-shed exosomes, HGF nanounit indirectly undermined the immune-resistant potency of exosomal PD-L1, causing the unleashed tumoricidal potential of cytotoxic T lymphocytes (CTLs). These sheltered T cells emitted vast quantities of inflammatory interferon-γ (IFN-γ), in turn downregulating the expression of tumor cellular cystine/glutamate transporters. This caused intracellular glutathione (GSH) depletion and lipid peroxidation, as a redox crisis that was compounded by enriched cellular iron from HGF NP, culminating in the potent induction of tumor ferroptosis. This immunotherapeutic tactic also improved responses to ICB therapy, adequate to eliminate lung metastatic lesions (Figure 2g,h). Likewise, we devised a phototheranostic metal-phenolic network (PFG MPN) via assembly of semiconductor polymer encapsulating GW4869 and Fe^3+^.^4^ Except for reducing the immunosuppression driven by tumor-shed exosomes and promoting cell ferroptosis, PFG MPN boosted photothermal efficacy for enhanced antitumor immunity. Collectively, by converting hypoimmunogenic tumor cells into potent in situ sources of tumor antigens and danger signals, or through dismantling checkpoint-mediated immunosuppression, these tumor-cell-targeted strategies provide the immunogenic substrates and stimulatory context required for the subsequent APC-mediated priming step of the CIC.

## 3 Targeting APCs

APCs, particularly dendritic cells (DCs) and macrophages, are indispensable regulators of the CIC, whose engagement dictates the functions of T lymphocytes.^7,8^ Once engaged, APCs primarily transmit two discrete yet functionally interdependent signal patterns for T-cell priming.^39,40^ The first signal arises from antigenic peptides bound to MHC molecules, allowing for specific antigenic recognition via the TCR.^41^ The second signal is mediated by antigen-nonspecific costimulatory molecules, such as CD80 and CD86 of the B7 family, which interact with CD28 on T lymphocytes to permit full activation.^42^ Both signals are indispensable, and omission of either abrogates effective T-cell immunity. Metal–ligand coordination nanomaterials, by virtue of the intrinsic immunostimulatory properties of metal ions and their unique ability to self-assemble with diverse adjuvant motifs, provide a unique advantage in amplifying these signals and strengthening the CIC.

### 3.1 Promoting Antigen Presentation

The commencement of antitumor T cell immunity depends on efficient tumor antigen recognition, which in turn requires effective antigen processing and presentation on MHC molecules.^41^ In general, endogenous antigenic motifs are subjected to presentation by MHC class I molecules to CD8^+^ T cell subsets, while exogenous antigens undergo presentation by MHC class II molecules to CD4^+^ T lymphocytes.^43^ Significantly, the presentation of exogenous antigenic components on MHC class I molecules, so-called cross-presentation, disrupts this traditional antigen-presentation pattern, mounting a cytotoxic T-cell-mediated response.^44^ In this way, a key challenge in devising next-generation cancer immunotherapeutics is to coordinate the requirements for adequate antigen supply and efficient antigen-presentation efficacy within APCs, especially cross-presentation.

Cross-presentation is predominantly mediated by DCs, whereas macrophages, despite their excellent phagocytic capacity, contribute minimally.^45^ This limitation is largely due to elevated lysosomal protease activity in macrophages, which rapidly degrades antigenic peptides and impairs cross-presentation.^46^ Based on this, together with considering that proteolysis is susceptible to pH changes, our team introduced a simple alkaline MgAl-based hydrotalcite (bLDH) that modulated lysosomal proteolytic activity in macrophages for improved antigen cross-presentation following RT (Figure 3a).^47^ Through impeding phagosomal acidification, bLDH nanoadjuvant compromised the proteolytic activity of phagolysosomal proteases, yielding MHC class I-dependent cross-presentation of engulfed tumor antigens within macrophages. This immunostimulatory effect was markedly amplified via coadministration of bLDH with cytosine-phosphate-guanosine (CpG) oligodeoxynucleotides that enhanced the phagocytic ability of macrophages (Figure 3b,c). These trained macrophages further enhanced natural killer (NK) cell cytotoxicity (Figure 3d) and cross-primed tumor-specific CD8^+^ T lymphocytes (Figure 3e), culminating in near-complete neoplasm obliteration (Figure 3f) that conferred a distinct survival benefit (Figure 3g).

**Figure 3:**
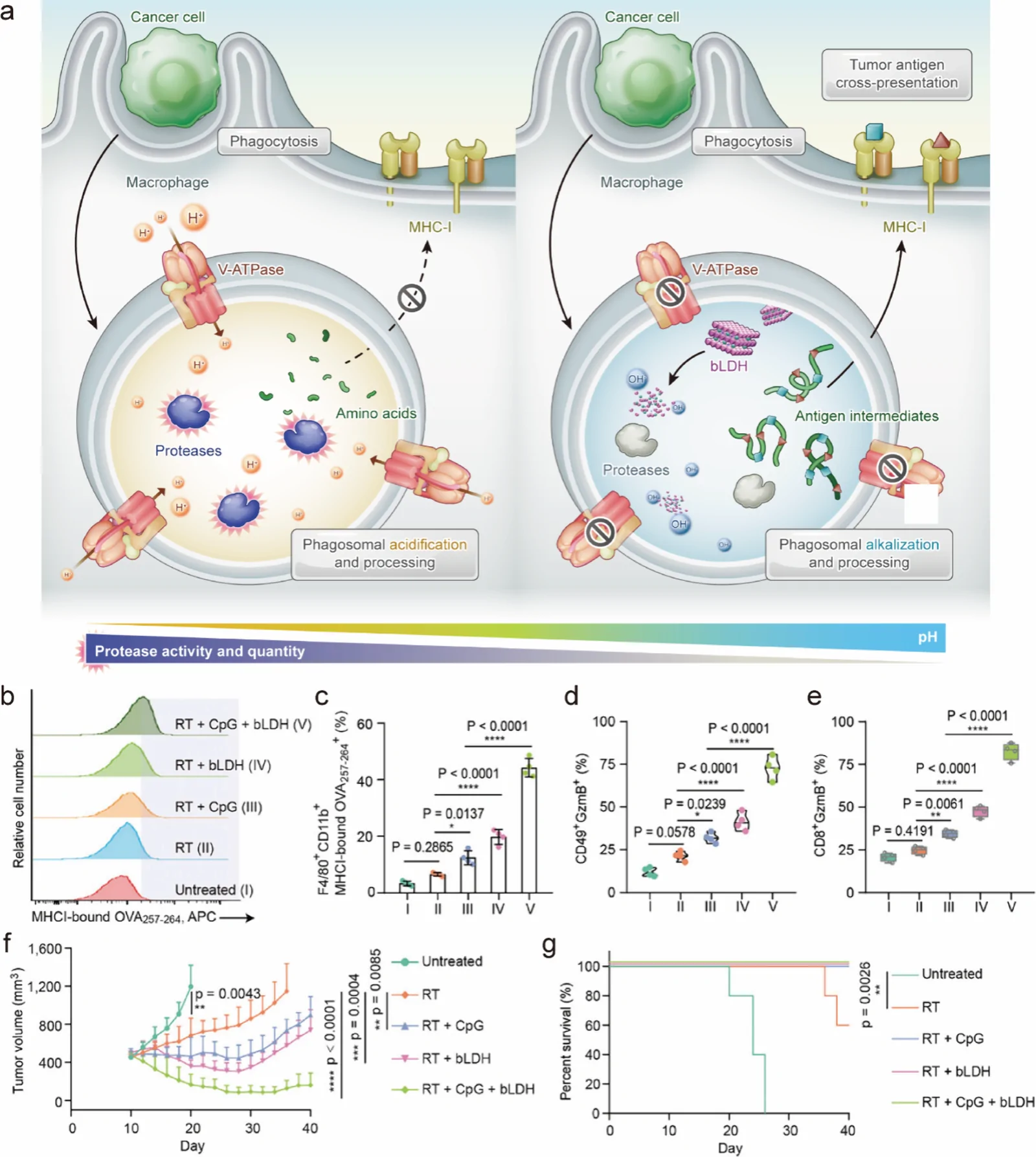
Immunotherapeutic mechanisms that strengthen antigen presentation of APCs. (a) Schematic illustration of alkaline bLDH-provoked antineoplastic immunity for enhanced antigen cross-presentation in macrophages. (b) Typical flow cytometric histograms and (c) quantified results exhibiting antigen cross-presentation of tumor-infiltrating macrophages. (d) The percentages of GzmB^+^ NK cells within tumor tissues. (e) Quantitative flow cytometric analysis of intratumoral GzmB^+^ T lymphocytes. (f) Tumor volumes of mice. (g) Kaplan–Meier survival curves of mice. Reproduced with permission from ref 47. Copyright 2025 Wiley-VCH.

### 3.2 Enhancing Costimulatory Signals

Following initial antigen recognition, costimulatory signals are an indispensable prerequisite for augmenting both the potency and endurance of antitumor T-cell immunity.^42^ In the absence of these signals, antigen-stimulated T cells become either functionally unresponsive or actively tolerized.^43^ Whereas the diversified immune-resistant networks within the TME can domesticate APCs for systematically reprogramming their phenotype and even driving profound abnormalities in their functionality. These abnormalities are notably manifested by the incompetence in upregulating the requisite costimulatory molecules.^48^ In this context, strategies that restore or enhance APC-mediated costimulation represent a rational approach to eliciting vigorous and durable T cell responses.

APCs are equipped with an extensive repertoire of recognition receptors prevailing within the innate immunity network, which are qualified for promoting their antineoplastic performance.^49^ Prior research has substantiated that certain metal ions participate in stimulating the various innate immune-related processes.^10,50^ Along this line, our group put forward an immunotherapeutic tactic that coupled this Mn^2+^-amplified innate signaling with RT sensitization, with the ultimate objective of potent antitumor T-cell immunity (Figure 4a).^51^ To this end, Mn^2+^ and radiosensitizer (NaGdF_4_:Nd@NaLuF_4_) were introduced to coordinate with PEG-polyphenol copolymer, eventually yielding a multifunctional metal-phenolic network designated as NaGdF_4_:Nd@NaLuF_4_@PEG-polyphenol/Mn (DSPM). Once targeted to tumor regions, pH-responsive DSPM nanoplatforms actively disintegrated, accompanied by the release of their payloads. Upon X-ray irradiation, the augmented RT expedited tumor cell death, concomitant with the massive leakage of dsDNA components into TME. This dsDNA-actuated STING signaling was in turn amplified by the coreleased Mn^2+^, which elicited a multifaceted immunostimulatory program that promoted DC maturation with the elevated expression of CD80 and CD86 (Figure 4b–d). Consequently, these activated DCs engaged in the priming of vigorous T-cell immunity capable of eradicating primary and metastatic lesions.

**Figure 4:**
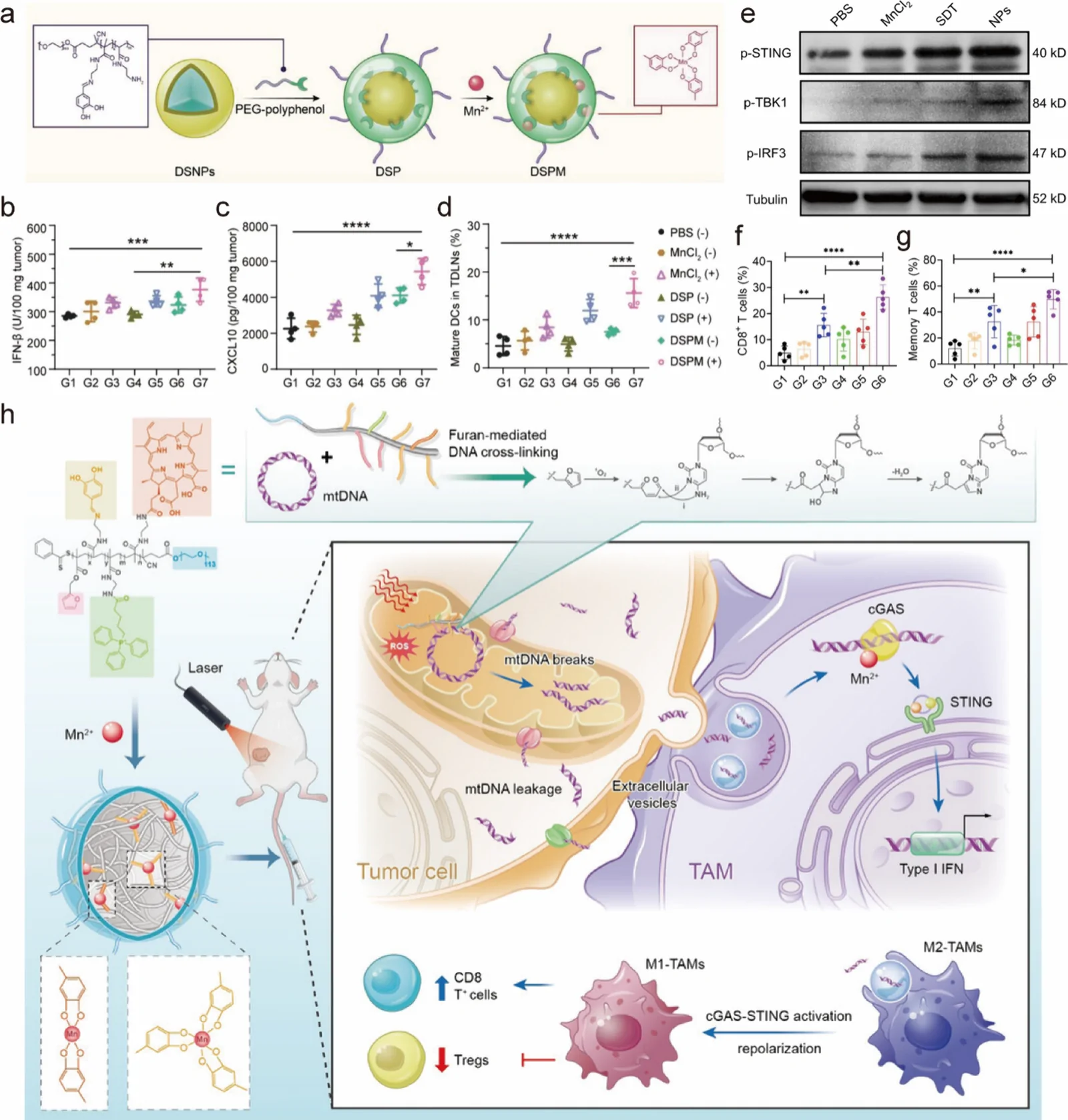
Immunotherapeutic tactics for heightening the expression of costimulatory signals on APCs. (a) Design and fabrication of DSPM nanoplatform. The level of (b) IFN-β and (c) CXCL10 within tumor regions. (d) Quantitative flow cytometric analysis of mature DCs within tumor-draining lymph glands. Reproduced with permission from ref 51. Copyright 2022 Wiley-VCH. (e) Western blot analysis of PIMS NP-mediated activation effects on the STING pathway. (f) Quantitative evaluation of tumor-infiltrating CD8^+^ T lymphocytes. (g) The proportions of memory T cells in the splenocytes. G1: PBS(−), G2: PBS­(+), G3: aPD-L1, G4: PIMS NP(−), G5: PIMS NP­(+), G6: PIMS NP combined with aPD-L1­(+). Reproduced with permission from ref 52. Copyright 2022 Elsevier. (h) Schematic exhibiting the action mechanism of PFM-induced antineoplastic immunity via stimulating STING pathway. Reproduced with permission from ref 54. Copyright 2024 Wiley-VCH.

Likewise, we designed a versatile metal-phenolic nanoadjuvant leveraging STING-dependent immunostimulation in alliance with sonodynamic therapy (SDT), substantially promoting DC maturation.^52^ This nanoadjuvant, termed PIMS, was synthesized by the self-assembly of Mn^2+^, a sonosensitizer (PEG-*b*-IR), a GSH inhibitor (sabutoclax), and a pH-responsive phenolic copolymer (PEG-*b*-Pho). The in situ liberation of Mn^2+^ fueled by the acidic TME, together with ultrasound-triggered leakage of plentiful DAMPs and tumor antigenic motifs, provided a dual stimulus for activating the STING pathway that propelled DC maturation (Figure 4e). This immuno-activating behavior further initiated a potent and long-lasting adaptive immunity, even improving the treatment sensitivity to ICB immunotherapy (Figure 4f,g). The combination tactic yielded a notable therapeutic benefit, as confirmed by the substantial elimination of primary and distal tumor lesions and pronounced inhibition of pulmonary metastasis.

Although macrophages are considered semiprofessional APCs, they can facilitate effective T-cell activation via upregulating costimulatory factors on their surface.^53^ Building on this concept, we proposed a macrophage-reprogramming strategy contingent on both the excessive mitochondrial DNA (mtDNA) damage- and Mn^2+^-driven activation of the STING signaling pathway (Figure 4h).^54^ For this purpose, our team utilized a novel amphiphilic phenolic copolymer modified with polyphenolic units, photosensitizers (Ce6), furan segments, and mitochondria-targeting moieties (triphenylphosphonium) to coordinate Mn^2+^, resulting in the formation of a metal-phenolic complex termed the PFM nanoplatform. The oxidant stress imposed by photodynamic therapy (PDT) and furan moiety-induced DNA cross-linking together triggered extensive damage and the subsequent release of mtDNA fragments. This tumor-derived mtDNA, coupled with free Mn^2+^, promoted re-education of M2-like macrophages toward M1 phenotypes, yielding a STING-dependent antitumor immune response. Taken together, these APC-directed platforms amplify both antigen presentation and costimulatory signaling, ensuring that primed tumor-specific T cells receive the two obligatory signals required for full activation, thereby directly feeding into the T cell effector phase of the CIC.

## 4 Targeting T Cells

Serving as the ultimate effector cells that carry out tumor cell killing and modulate the magnitude of the entire immune response, T lymphocytes dictate the fate of antitumor immunity through the efficiency of their activation, trafficking, infiltration, and cytolytic activity.^7,8^ Whereas, T-cell functions are frequently compromised by tumors via the concerted exploitation of multiple mechanisms that operate at one or more of these critical steps within the CIC.^55^ Emerging immunotherapeutic modalities represented by metal–ligand coordination nanomedicines exert their immune-activating effects by employing therapeutic strategies purposefully designed to dismantle this tumor-mediated immunosuppression, culminating in the reinitiation or potentiation of the CIC.

### 4.1 Strengthening T-Cell Activation

The generation of effective antitumor immunity is substantially dependent on the activation status of T lymphocytes, in particular that of the cytotoxic T-cell population.^56,57^ This activation requires coordinated interactions with APCs in the nearby lymph nodes (LNs), a process that is highly susceptible to the immunosuppressive microenvironment.^56^ Prior work has illustrated that metal ions can directly modulate T-cell activation, functioning either as structural scaffolds that stabilize TCR-mediated complexes or as modulators of downstream effector programs.^9^ For example, Mg^2+^ enhances CTL effector activity by amplifying TCR stimulation and enhancing downstream signal cascades.^11^ Furthermore, Fe^3+^-driven ROS generation can promote CTL activation in a nuclear factor of activated T cells (NFAT)-dependent manner.^58^ These data underscore the potential of metal-containing immunoadjuvants to fine-tune T cell activation signals, reinforcing the cytolytic activity of CTLs.

Considering the unique structure and constitution of layered double hydroxides (LDHs), we devised a postoperative flex-patch that integrated magnesium–iron-based LDHs and PD-1 antibody to reinvigorate CTLs within sentinel LNs (Figure 5a).^1^ This patch augmented TCR-mediated signaling cascades for further reinforcing downstream T-cell activation programs propelled by metal ions (Figure 5b–d). The combined application of LDH nanoadjuvant and ICB therapy significantly boosted tumor-specific CTL responses within metastatic sentinel LNs and established systemic immune memory, leading to the sustained surveillance against postsurgical breast cancer recurrence.

**Figure 5:**
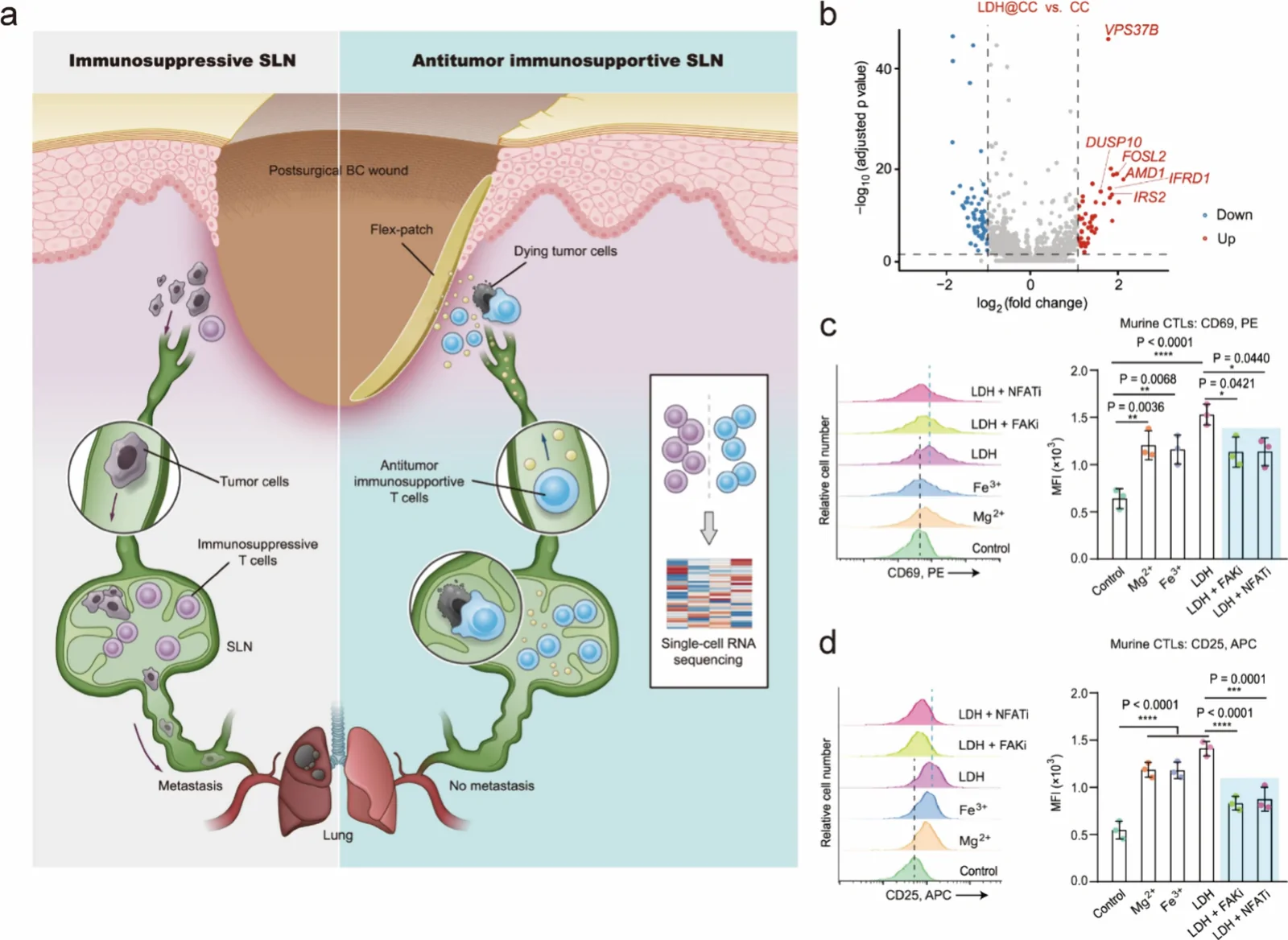
Design strategies for stimulation of T-cell activation. (a) Therapeutic mechanism illustration of LDH-delivering flex-patch-mediated antineoplastic immunity via revitalizing CTLs within sentinel LNs. (b) Volcano plots exhibiting differentially expressed genes in activated T lymphocytes. (c) CD69 expression on murine T cells. (d) The percentages of CD25^+^ T lymphocytes. Reproduced with permission from ref 1. Copyright 2023 Springer Nature.

Apart from direct intervention on T-cell activation signaling networks, we also proposed a tumor metabolic-reprogramming strategy that preserved energy for CD8^+^ T lymphocytes for a favorable antineoplastic battlefield.^59^ An HA-modified metal-phenolic nanotherapeutic (HPP-Ca@GSK) was fabricated via the simple assembly of lactate dehydrogenase A (LDHA) inhibitor (GSK), sonosensitizer (PEG-IR780), PEG-phenol, CaCl_2_, and HA-catechol. By inhibiting LDHA, these nanomedicines restrained aerobic glycolysis within tumor cells, generating a metabolic milieu enriched with glucose and depleted of lactate. Mechanistically, this glucose-enriched, lactate-depleted milieu destabilized intratumoral regulatory T (T_reg_) cells through two complementary routes. Removal of lactate supply deprived T_reg_ cells of the preferential metabolite that maintained their suppressive function and proliferation within tumors.^60^ Moreover, the glucose-enriched environment was detrimental to T_reg_ cells, since high-glucose conditions impaired the function and long-term stability of T_reg_ cells, while simultaneously supporting the glucose-dependent bioenergetic and biosynthetic demands of proliferating CD8^+^ CTLs.^61,62^ Coupled with robust induction of ICD, HPP-Ca@GSK nanotherapeutics further empowered the antitumor activities of CD8^+^ T lymphocytes.

### 4.2 Accelerating T-Lymphocyte Trafficking and Infiltration

For CTLs to exert tumor-specific cytotoxicity, they must migrate from lymphoid organs into the bloodstream, extravasate across the vascular endothelium, and infiltrate the tumor bed.^7,8^ Disruption of any step renders the CIC ineffective. The efficiency of T-cell trafficking and infiltration is governed by complex interactions among chemotactic signals, metabolic conditions, and immunosuppressive networks within the TME.^7^ Overcoming these barriers is essential for sustaining effective antitumor immunity.

Inspired by the capacity of interferon signaling to recruit T cells and enhance their intratumoral infiltration, our group developed a metal-phenolic network that triggered intracellular type I interferon (IFN-I) generation for enhanced T-cell-mediated CIC (Figure 6a).^63^ Specifically, X-ray-sensitive W^6+^ ions were introduced to self-assemble with DNA methyltransferase inhibitor (5-Aza) and histone deacetylase suppressor (ITF-2357) to construct the ultimate PWAI nanoplatform. Mechanistically, this dual epigenetic intervention restored the IFN-I response that was transcriptionally silenced in the MYC-driven tumors. Specifically, 5-Aza reversed promoter hypermethylation to derepress interferon-regulatory genes, whereas ITF-2357 promoted histone acetylation to counteract MYC-mediated suppression of IFN-I signaling-associated gene transcription. These combined actions reactivated the intracellular IFN-I program (Figure 6b,c), while potentiating the W^6+^-driven tumoricidal efficacy of RT. Beyond itself, IFN-I instigated a downstream program characterized by upregulated C-X-C motif chemokine ligand (CXCL)­9, CXCL10, and CXCL11 and consequent C-X-C motif chemokine receptor (CXCR)­3 signaling on activated T cells.^64^ Collectively, chemotactic cues collectively guided CD8^+^ and CD4^+^ T-cell recruitment to tumors (Figure 6d,e). Likewise, we designed additional nanoadjuvants such as PCM NPs, synthesized through the coordination of Cu^2+^ and PEG-polyphenol-Ce6.^65^ By inducing IFN-I signaling, PCM nanotherapeutics promoted deep interstitial penetration of immunostimulatory T cells into tumor lesions.

**Figure 6:**
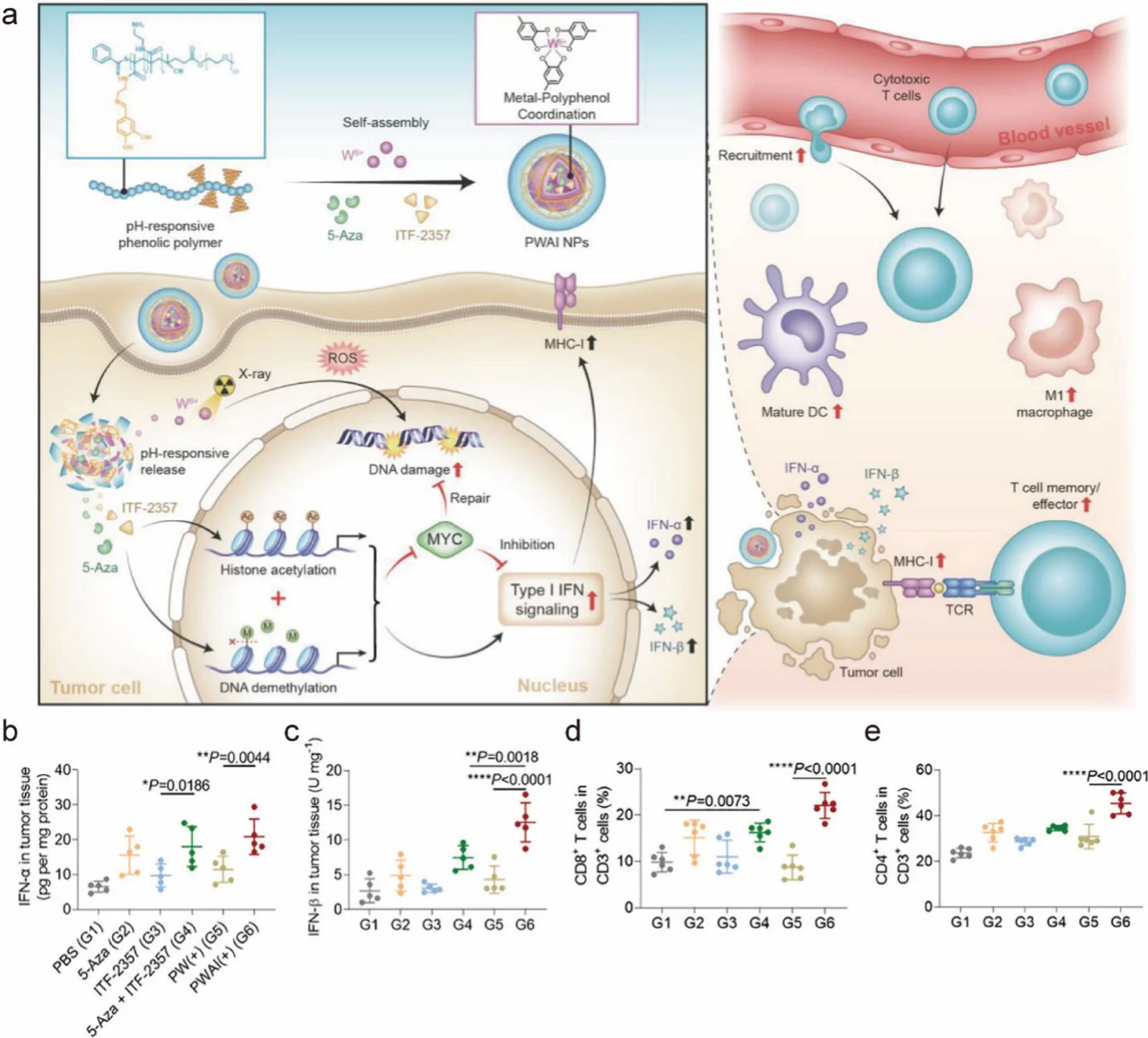
Immunotherapeutic tactics for accelerating T-lymphocyte trafficking and infiltration. (a) Schematic illustration exhibiting PWAI NP-evoked antineoplastic immunity via IFN-driven T-cell recruitment and tumoral infiltration. (b) IFN-α and (c) IFN-β levels within tumor tissues. (d) Quantitative analysis of intratumoral CD3^+^CD8^+^ T lymphocytes. (e) The percentages of tumor-infiltrating CD3^+^CD4^+^ T cells. Reproduced with permission from ref 63. Copyright 2024 Wiley-VCH.

### 4.3 Potentiating T-Cell Cytotoxicity

T-cell-mediated cytotoxicity not only eliminates tumor cells but also reconnects the effector phase to the CIC initiation through the release of DAMPs and tumor antigens.^8^ This feedback loop transforms antitumor immunity from a single attack into a sustained, self-amplifying cascade and serves as a foundation for immunological memory. Upon reexposure to cognate tumor antigens, memory T cells can rapidly mount renewed tumor-specific responses, perpetuating the CIC and securing extended protection against tumor reemergence.^66^

To boost T-cell tumor-killing capacity, we synthesized an immunotherapeutic nanoeditor (Figure 7a).^67^ Amphipathic PEG polymers modified with the choline analogue 2 methacryloyloxyethyl phosphorylcholine (MPC) units and phenol moieties were assembled with Mg^2+^ and β-site amyloid precursor protein-cleaving enzyme 1 (BACE1) inhibitor (MK-8931) to customize Mg^2+^@MK-8931@MPP nanoeditor. Following MK-8931 release, these nanoeditors repolarized immunosuppressive M2-like macrophages toward proinflammatory M1 phenotypes, leading to ameliorative recruitment of CTLs and weakened infiltration of T_reg_ cells. Concurrently, Mg^2+^ strengthened TCR downstream signaling, enhancing the effector functions of tumor-infiltrating CTLs and promoting secretion of antineoplastic cytokines and cytotoxic proteins (Figure 7b). Significantly, blockage of immune-resistant NK1.1 on CTLs prolonged their cytolytic activity (Figure 7c), achieving obvious clearance of intracranial tumor lesions (Figure 7d).

**Figure 7:**
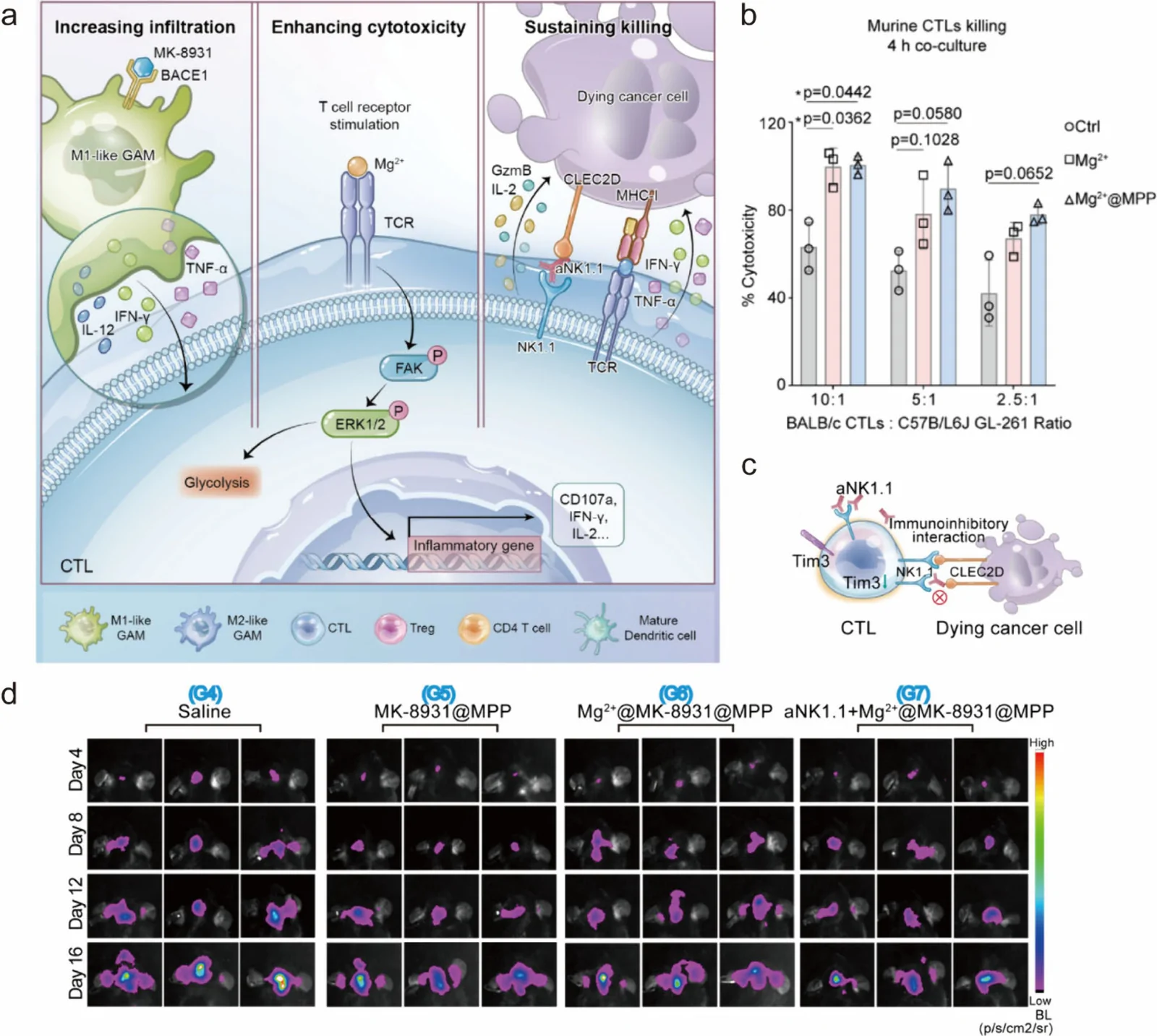
Immunotherapeutic mechanisms that augment the tumoricidal capabilities of T cells. (a) Schematic diagram illustrating the tumoricidal effects of Mg^2+^@MK-8931@MPP nanoeditor against intracranial glioma. (b) In vitro cytotoxicity of murine T lymphocytes. (c) Graphical illustration of aNK1.1-provoked sustained T-cell cytotoxicity. (d) Representative in vivo bioluminescence imaging of mice. Reproduced with permission from ref 67. Copyright 2025 American Chemical Society.

## 5 Conclusion

Metal–ligand coordination nanomaterials have been identified as versatile metalloimmunotherapeutics capable of engaging multiple nodes of the CIC. In this Account, we systematized these nanomedicines according to their three principal CIC targets, including tumor cells, APCs, and T cells, establishing a mechanistic framework that clarifies how metal coordination chemistry can be rationally exploited beyond conventional drug delivery. This target-centric perspective reveals design principles that link compositional tunability to immunomodulatory function, underscoring the dual role of metal ions as both structural scaffolds and innate immune cues.

Despite substantial preclinical advances, the clinical translation of CIC-tailored metal coordination nanomaterials remains at an early stage. Key barriers include incomplete understanding of in vivo pharmacokinetics and metal-ion biodistribution, potential cumulative metal toxicity, lack of standardized long-term biosafety assessments, and the difficulty of achieving reproducible large-scale synthesis under pharmaceutical quality controls. These barriers are particularly acute for metal-coordination nanomaterials because of the dynamic nature of the coordination bond, wherein metal speciation, oxidation state, and ligand-exchange behavior in biological fluids can vary substantially between formulations and even batches, complicating pharmacokinetic predictability and safety assessment. Addressing these gaps demands systematic profiling of structure-pharmacokinetic relationships, clinically relevant toxicity benchmarks, and scalable modular manufacturing approaches with rigorous batch-to-batch consistency. In particular, we propose that high-throughput screening of metal–ligand libraries, combined with in situ characterization technologies (e.g., correlative spectroscopic and imaging approaches that track metal speciation, subcellular fate, and immune engagement), should be harnessed to establish standardized biosafety assessment benchmarks. Such an integrated platform would enable systematic profiling of metal-ion dose–response relationships, cumulative toxicity thresholds, and clearance kinetics across a wide chemical space, while in situ readouts would link molecular-level metal speciation to organ-level pharmacokinetics and immunotoxicity. The resulting benchmarks would support the rational preselection of nanotherapeutics with optimal safety windows and, in tandem with scalable manufacturing, accelerate their transition from laboratory platforms to clinically viable cancer therapeutics.
